# Anemia and associated factors among adolescent girls and boys at 10–14 years in rural western China

**DOI:** 10.1186/s12889-021-10268-z

**Published:** 2021-01-26

**Authors:** Zhonghai Zhu, Christopher R. Sudfeld, Yue Cheng, Qi Qi, Shaoru Li, Mohamed Elhoumed, Wenfang Yang, Suying Chang, Michael J. Dibley, Lingxia Zeng, Wafaie W. Fawzi

**Affiliations:** 1 Department of Epidemiology and Biostatistics, School of Public Health, Xi’an Jiaotong University Health Science Center, No.76, Yanta West Road, Xi’an, Shaanxi 710061 People’s Republic of China; 2 Department of Global Health and Population, Harvard T.H. Chan School of Public Health, Boston, MA USA; 3 Department of Nutrition and Food Safety Research, School of Public Health, Xi’an Jiaotong University Health Science Center, Xi’an, Shaanxi 710061 People’s Republic of China; 4 National Institute of Public Health Research (INRSP), BP. 695, Nouakchott, Mauritania; 5 Department of Obstetrics and Gynecology, The First Affiliated Hospital of Xi’an Jiaotong University, Xi’an, Shaanxi People’s Republic of China; 6 United Nations Children’s Fund, China Office, Beijing, 100600 People’s Republic of China; 7 The Sydney School of Public Health, Faculty of Medicine, The University of Sydney, Sydney, NSW Australia; 8 Key Laboratory of Environment and Genes Related to Diseases, Xi’an Jiaotong University, Xi’an, 710061 Shaanxi People’s Republic of China

**Keywords:** Rural western China, Adolescent, Anemia, Associated factors, Puberty development, Dietary intake, Social determinant

## Abstract

**Background:**

Evidence on anemia and associated factors among young adolescent girls and boys in rural western China is limited.

**Methods:**

We used data from a follow-up study of adolescents (10–14 years) born to women who participated in a randomized trial of antenatal micronutrient supplementation in western China. Anemia was defined by World Health Organization standards. Logistic regression was used to examine the factors associated with anemia.

**Results:**

The overall prevalence of anemia was 11.7% (178/1517). Female adolescents were 1.73 (95% CI 1.21, 2.48) times more likely to have anemia as compared to males. Adolescents whose mothers had completed high school were 0.35 (95% CI 0.13, 0.93) times less likely to be anemic, compared to those of whom had < 3 years of formal education. Household wealth was also inversely associated with anemia. The association of puberty status with anemia was modified by adolescent sex (*P*-value for interaction was 0.04); males with greater than mild pubertal development had reduced odds (OR 0.35, 95% CI 0.15, 0.83) of anemia while there was no association among females (OR 0.72, 95% CI 0.29, 1.78). Consumption of flesh foods (OR 0.58, 95% CI 0.38, 0.89), eggs (OR 0.60, 95% CI 0.38, 0.93), and having a meal frequency of three times or more per day (OR 0.68, 95% CI 0.48, 0.96) were also associated with a lower likelihood of anemia.

**Conclusions:**

Anemia was a mild public health problem among young adolescents in rural western China. Nutritional and social determinants were identified as predictors, warranting interventions to reduce the risk of anemia among this critical age group.

**Supplementary Information:**

The online version contains supplementary material available at 10.1186/s12889-021-10268-z.

## Background

Adolescence is a critical period of growth, reproductive maturation, and developmental transitions which requires increased nutritional intake and therefore makes adolescents more vulnerable to nutritional deficiencies [1]. Nearly 90% of adolescents live in low- and middle-income countries (LMICs) where undernutrition, including anemia and micronutrient deficiencies, remains public health problems [2]. Anemia in adolescence may cause a wide range of functional consequences across the life course, including reduced resistance to infection, impaired physical performance and neurodevelopment, and suboptimal schooling outcomes [3, 4].

Globally, many prior studies have investigated the prevalence of anemia and associated factors among adolescent girls [5]; these studies suggest that, the prevalence of anemia differs by country and region with estimates ranging from 5.3% in high-income country settings to over 50.0% in some LMICs [6, 7]. Further, there is limited data that shows on anemia prevalence among boys in LMICs. One study from India reported that the prevalence of anemia among adolescent boys was as high as the prevalence among girls at 50.0 and 56.5%, respectively [8]. In addition, studies have reported inconsistent associations between pubertal development and adolescent anemia among boys and girls [9].

Multiple studies have reported that factors associated with anemia differ for adolescents as compared to adults [10, 11]. Additionally, most of growth gained during adolescence occurs at early adolescence (age 10–14 years) defined by the United Nations [12]. Studies have also reported that the prevalence of anemia may differ between early adolescents and older adolescents (age 15–19 years) [7, 13, 14]. Given that iron deficiency may be a key factor for anemia [15] and that early adolescence is a critical period of diet transition from childhood to adolescence [16, 17], improving adolescent dietary intake may be a reasonable approach to reduce anemia [18]. However, studies have reported inconsistent results on the associations of eggs, dairy products, and heme-iron containing food sources with anemia [13, 19–21].

China has experienced rapid development and urbanization in the past few decades that has corresponded with major changes in dietary patterns [22]. Recent data on Chinese adolescent anemia has been obtained from the national survey [23–25], providing an incomplete examination of the epidemiology of anemia in this age group, e.g., missing to examine influencing factors. The prevalence of anemia among Chinese adolescents aged 12 and 14 years was 9.6 and 8.4%, respectively, and ranged from 2.0% in Beijing to 24.1% in Hainan [25]. These results suggest that despite some improvement of wealth and diets in rural China, adolescents may still have a high burden of anemia due to the suboptimal intake of iron-rich foods, vitamins, and other micronutrients [26].

In this paper, we analyzed data from adolescents in rural western China to assess factors associated with anemia among young adolescents aged 10–14 years. These data are intended to inform intervention strategies for this critical age group.

## Methods

### Study design and participants

This study was a secondary analysis of data from a follow-up study of adolescents born to women who participated in a cluster-randomized controlled trial of prenatal micronutrient supplementation. The details of the original trial and the adolescent follow-up study were described elsewhere [27, 28].

Briefly, all pregnant women in villages from two counties in rural western China, where an anemia prevention program for adolescents was not implemented, were randomly assigned to take a daily capsule of folic acid, folic acid plus iron, or multiple micronutrients between 2002 and 2006. A total of 4488 singleton live births were eligible for long-term follow-up. We followed 1517 adolescents aged 10–14 years between June and December 2016. Due to the rapid urbanization, moving out of the study area was the primary reason for loss to follow-up.

Venous blood samples were collected from each participant by a nurse in a local hospital and were immediately tested for hemoglobin concentration using an automated hematology analyzer (BC-3000Plus, mindray).

Anthropometric measurements were assessed, including height and weight. After removal of shoes and heavy clothing, standing height was measured to the nearest 0.1 cm using a steel strip stadiometer (SZG − 210, Shanghai JWFU Medical Apparatus Corporation), and body weight was measured to the nearest 0.1 kg (BC-420, Tanita Corporation, Tokyo, Japan). Two measurements for height were performed and if a discrepancy occurred, repeated measures were taken until consensus obtained. Body mass index (BMI) was calculated as body weight divided by height squared (kg/m^2^). BMI-for- age and sex Z-score (BAZ) and height-for-age and sex Z-score (HAZ) were used to classify the nutritional status as thinness (BAZ < -2 SD) or stunting (HAZ < -2 SD), and overweight (BAZ > 1 SD) or stature above average (HAZ > 1 SD) according to the World Health Organization (WHO) growth standards [29].

Puberty was assessed with the Tanner scale by rigorously trained medical graduate students using standard procedures. The Tanner scale included five-level assessment of developmental stages of the scrotum and pubic hair in males, and breasts and pubic hair in females, respectively [30, 31]. Specifically, among females, both pubic hair and breasts showing the characteristics of Stage 1 indicated pre-puberty; both pubic hair and breasts showing the characteristics of Stage 2 indicated mild stages of puberty; either pubic hair or breast that showed the characteristics of Stage 3 or beyond indicated above mild stage (Due to scarce data, Stages 3, 4 and 5 were merged in the present study). For boys, the frequencies of Tanner stage 1 to 5 were 182 (21.0%), 420 (48.5%), 182 (21.0%), 78 (9.0%) and 4 (0.5%), respectively. As for girls, the corresponding frequencies were 78 (12.2%), 226(35.2%), 286 (44.6%), 52 (8.1%) and 0 (0.0%), respectively. Similar cut-off criteria were applied to the developmental stages of the scrotum and pubic hair in males.

A structured questionnaire was used to collect information on socioeconomic status and adolescent disease history by a trained public health graduate student. Adolescents’ primary caregivers provided all information except for dietary intake which was directly collected from the adolescents. Adolescent dietary intake was collected using a food group-based frequency questionnaire. As an example, adolescents were asked “How often do you consume flesh foods such as meat, poultry, and fish, i.e., almost never, almost 1/month, 1-3/month, 1/week, 2-4/week, 5-6/week, and ≥1/day?”. Consumption of food groups including beans, dairy products and egg, and meal frequency in 24 h were similarly collected. We then transformed these frequencies into the estimated number of times an adolescent consumed each food group per day and classified intake into tertiles within the sample. Socioeconomic status data included parental age, education, occupation, and household wealth. Adolescent health history was defined as whether the adolescent had seen a doctor in the prior 2 weeks. Household wealth index was constructed by principal component analysis of 17 household assets and the ownership of goats, cattle, horses, and poultry, which were then categorized into tertiles that indicated low-, middle- and high-income households [32].

### Statistical analysis

Anemia was defined and adjusted for age as recommended by WHO [33], and was defined by hemoglobin concentrations < 115 g/L among adolescents 10–11 years old and < 120 g/L among adolescents 12–14 years old. In terms of severity, mild anemia was defined as a hemoglobin concentration between 110 and 114 g/L among adolescents aged 10–11 or between 110 and 119 g/L among adolescents aged 12–14. Moderate and severe anemia was defined by a hemoglobin concentration of between 80 and 109 g/L and lower than 80 g/L, respectively.

Counts/percentages for categorical variables and means±standard deviations (SDs) for continuous variables were used to describe characteristics of the study population. Bivariate and multivariable logistic regression models were performed to examine factors associated with anemia including socioeconomic status, randomized regimens during pregnancy, adolescent age, sex, nutrition status, disease history, stages of puberty and dietary intake. All potential factors were considered in the multivariable analyses. We also examined influencing factors of continuous hemoglobin concentrations using generalized linear models. Finally, we performed stratified analysis by sex with other variables included in the model. *P* values for interactions by sex were estimated from likelihood ratio tests comparing models including and excluding interaction terms between sex and factors.

Data were analyzed using STATA 15.0 (StataCorp, College Station, TX, USA). A two-sided *P* value < 0.05 was considered statistically significant.

## Results

A total of 1517 adolescents age 10–14 years were interviewed, had hemoglobin data, and were included in the analyses. Table 1 presents the socioeconomic and nutritional characteristics of the study participants. The mean age of adolescents, their mothers and fathers were 11.8 ± 0.9, 37.3 ± 4.4 and 39.5 ± 4.1 years, respectively. Of adolescents, 873 (57.6%) were female. Majority of mothers and fathers had secondary education or higher. More than half of mothers were farmers, but only 34.4% of fathers were farmers.

**Table 1:** Background characteristics and anemia status among adolescents in rural Western China, 2016^a^

Parental characteristics	Total/n(%)	Adolescent characteristics	Total/n(%)
N	1517 (100.0)	N	1517 (100.0)
Maternal age/years^b^		Adolescent age/years (Mean ± SD)	11.8 (0.9)
Mean (SD)	37.3 (4.4)	10	73 (4.8)
Q1: ≤35	642 (42.3)	11	538 (35.5)
Q2: 36–39	408 (26.9)	12	547 (36.1)
Q3: ≥40	467 (30.8)	13–14 (only two children were 14)	359 (23.7)
Maternal education		Sex	
< 3 years	87 (5.8)	Male	873 (57.6)
Primary	433 (28.6)	Female	644 (42.5)
Secondary	757 (50.1)	Hemoglobin concentrations (g/L, Mean ± SD)	133.0 (14.9)
High school+	235 (15.5)	BMI for age z score	
Maternal occupation		Thinness (<−2SD)	82 (5.5)
Farmer	923 (60.9)	Normal weight	1202 (80.0)
Others	593 (39.1)	Overweight (>1SD)	219 (14.6)
Paternal age/years^b^		Height for age z score	
Mean (SD)	39.5 (4.1)	Stunting (<−2SD)	32 (2.1)
Q1: ≤37	571 (37.8)	-2 to 1 SD	1184 (78.1)
Q2: 38–41	477 (31.6)	Stature above average (>1SD)	300 (19.8)
Q3: ≥42	464 (30.7)	Whether having an illness in last 2 weeks	
Paternal education		Yes	590 (39.0)
< 3 years	19 (1.3)	No	923 (61.0)
Primary	218 (14.4)	Puberty development^c^	
Secondary	894 (59.1)	Pre-puberty	260 (17.2)
High school+	383 (25.3)	Mild	646 (42.8)
Paternal occupation		Above mild	602 (39.9)
Farmer	507 (34.4)	Times of consuming flesh foods per day/Mean (SD)^d^	0.23 (0.28)
Others	968 (65.6)	Q1 (< 0.03)	374 (24.7)
Household wealth		Q2 (0.03–0.14)	523 (34.5)
Low	525 (34.6)	Q3 (> 0.14)	620 (40.9)
Medium	446 (29.4)	Times of consuming beans per day/Mean (SD)^d^	0.29 (0.33)
High	546 (36.0)	Q1 (< 0.03)	512 (34.0)
Randomized regimens		Q2 (0.03–0.43)	723 (48.0)
Folic acid	529 (34.9)	Q3 (> 0.43)	270 (17.9)
Iron/folic acid	502 (33.1)	Times of consuming dairy products per day/Mean(SD)^d^	0.33 (0.38)
Multiple micronutrients	486 (32.0)	Q1 (< 0.03)	583 (38.7)
		Q2 (0.03–0.43)	54 (30.5)
		Q3 (> 0.43)	36 (20.3)
		Times of consuming egg per day/Mean(SD)^d^	0.38 (0.37)
		Q1 (< 0.07)	526 (34.9)
		Q2 (0.07–0.43)	564 (37.4)
		Q3 (> 0.43)	419 (27.8)
		Meal frequency in 24 h	
		Two times	663 (44.2)
		Three times and four times^e^	836 (55.8)

^a^Data are missing for maternal education (*n* = 5), maternal occupation (*n* = 1), paternal education (*n* = 3), paternal occupation (*n* = 42), BMI for age z score (*n* = 14), height for age z score (*n* = 1), high protein-based food (*n* = 4), puberty development (*n* = 9), beans (*n* = 2), dairy products (*n* = 2), egg (*n* = 8), and meal frequency (*n* = 18)

^b^Parents’ age was categorized by its tertiles

^c^Puberty development was defined by the Tanner stages

^d^The frequency of consuming foods was converted into continuous variables, namely times per day, which were then categorized by its tertiles. Flesh foods included meat, poultry and fish

^e^Only 14 (0.9%) adolescents had a meal frequency of four times per day

The prevalence of thinness and stunting was 5.5 and 2.1%, respectively, while the prevalence of overweight and stature above average was 14.6 and 19.8%, respectively. Puberty onset had occurred among 82.8% of participants and 42.8% were in the mild stage of pubertal development. Regarding dietary intake, the frequencies of high protein-based foods consumptions including flesh foods, beans, dairy products, and eggs were fairly low and ranged from 0.23 to 0.38 times per day. Nearly half (44.2%) of the adolescents reported to have a meal frequency of two times per day.

### Factors associated with adolescent anemia

The average hemoglobin concentration was 133.0 ± 14.9 g/L. In total, age-adjusted anemia was found among 178 (11.73%) adolescents. Of these anemic adolescents, 93 (52.2%) were mild, 82 (46.1%) were moderate, while only 3 (1.7%) were severely anemic.

Unadjusted and multivariable analyses of factors associated with adolescent anemia are presented in Table 2. Adolescent girls were 1.73 (95% confidence interval [CI] 1.21, 2.48) times more likely to have anemia as compared to boys. We also found that the odds (odds ratio [OR] 0.50, 95% CI 0.29, 0.87) of anemia were lower in adolescents who were above mild stages of puberty development as compared to those in pre-puberty. Compared with adolescents whose mothers had < 3 years of formal education, those whose mothers completed education beyond high school were 0.35 (95% CI 0.13, 0.93) times less likely to be anemic. Adolescents from high-income households relative to those from low-income households were also less likely to be anemic, with an adjusted OR of 0.55 (95% CI 0.34, 0.88).

**Table 2:** Factors associated with adolescent anemia in rural western China, 2016 (*N* = 1517)

	Anemia/n(%)	Unadjusted			Adjusted^a^			*P* values for trend^a^
		OR	95% CI	*p* values	OR	95% CI	*p* values	
Maternal age/years^b^								0.50
Q1: ≤35	84 (13.1)	1.00			1.00			
Q2: 36–39	45 (11.0)	0.82	0.56, 1.21	0.32	0.85	0.53, 1.34	0.47	
Q3: ≥40	49 (10.5)	0.78	0.54, 1.13	0.19	0.89	0.46, 1.69	0.71	
Maternal education								0.07
< 3 years	17 (19.5)	1.00			1.00			
Primary	61 (14.1)	0.68	0.37, 1.22	0.20	0.60	0.31, 1.15	0.13	
Secondary	89 (11.8)	0.55	0.31, 0.97	0.04	0.56	0.28, 1.12	0.10	
High school+	11 (4.7)	0.20	0.09, 0.45	< 0.001	0.35	0.13, 0.93	0.04	
Maternal occupation								–
Farmer	130 (14.1)	1.00			1.00			
Others	48 (8.1)	0.54	0.38, 0.76	< 0.001	0.81	0.53, 1.22	0.31	
Paternal age/year^b^								0.42
Q1: ≤37	71 (12.4)	1.00			1.00			
Q2: 38–41	58 (12.2)	0.97	0.67, 1.41	0.89	0.97	0.62, 1.51	0.89	
Q3: ≥42	49 (10.6)	0.83	0.56, 1.22	0.35	0.71	0.36, 1.43	0.34	
Paternal education								0.88
< 3 years	4 (21.1)	1.00			1.00			
Primary	32 (14.7)	0.65	0.20, 2.07	0.46	0.57	0.16, 1.97	0.37	
Secondary	114 (12.8)	0.55	0.18, 1.68	0.29	0.63	0.18, 2.13	0.45	
High school+	28 (7.3)	0.30	0.09, 0.95	0.04	0.60	0.16, 2.20	0.44	
Paternal occupation								
Farmer	72 (14.2)	1.00			1.00			
Others	98 (10.1)	0.68	0.49, 0.94	0.02	0.87	0.59, 1.27	0.46	
Household wealth					0.78	0.63, 0.98		0.03
Low	75 (14.3)	1.00			1.00			
Medium	70 (15.7)	1.12	0.78, 1.59	0.54	1.17	0.80, 1.72	0.38	
High	33 (6.0)	0.39	0.25, 0.59	< 0.001	0.55	0.34, 0.88	0.01	
Randomized regimens								
Folic acid	55 (10.4)	1.00			1.00			
Iron/folic acid	62 (12.4)	1.21	0.83, 1.79	0.32	1.10	0.72, 1.66	0.68	
Multiple micronutrients	61 (12.6)	1.24	0.84, 1.82	0.28	1.27	0.85, 1.91	0.25	
Adolescent age					1.28	1.02, 1.62		0.04
10	6 (8.2)	1.00			1.00			
11	55 (10.2)	1.27	0.53, 3.07	0.59	1.01	0.40, 2.54	0.98	
12	71 (13.0)	1.67	0.70, 3.98	0.25	1.48	0.58, 3.77	0.41	
13–14	46 (12.8)	1.64	0.67, 4.00	0.28	1.64	0.61, 4.42	0.33	
Sex								
Male	85 (9.7)	1.00			1.00			
Female	93 (14.4)	1.56	1.14, 2.14	0.01	1.73	1.21, 2.48	0.003	
Height for age z score								0.23
Stunting (<−2SD)	7 (21.9)	1.89	0.80, 4.44	0.68	1.20	0.46, 3.13	0.71	
-2 to 1 SD	153 (12.9)	1.00			1.00			
Above average (>1SD)	18 (6.0)	0.43	0.26, 0.71	0.43	0.71	0.40, 1.24	0.22	
Whether having an illness in the last 2 weeks								
Yes	81 (13.7)	1.00			1.00			
No	95 (10.3)	0.72	0.53, 0.99	0.04	0.76	0.54, 1.06	0.10	
Puberty development^c^					0.70	0.53, 0.93		0.01
Pre-puberty	40 (15.4)	1.00			1.00			
Mild	71 (11.0)	0.68	0.45, 1.03	0.07	0.64	0.40, 1.01	0.06	
Above mild	66 (11.0)	0.68	0.44, 1.03	0.07	0.50	0.29, 0.87	0.01	
Times of consuming flesh foods per day (Mean/SD)^d^								0.11
Q1 (Lowest)	62 (16.6)	1.00			1.00			
Q2	54 (10.3)	0.58	0.39, 0.86	0.01	0.58	0.38, 0.89	0.01	
Q3 (Highest)	62 (10.0)	0.56	0.38, 0.82	0.003	0.72	0.48, 1.08	0.11	
Times of consuming beans per day (Mean/SD)^d^								0.48
Q1 (Lowest)	62 (12.1)	1.00			1.00			
Q2	94 (13.0)	1.08	0.77, 1.53	0.64	1.20	0.83, 1.73	0.34	
Q3 (Highest)	22 (8.2)	0.64	0.39, 1.07	0.09	0.75	0.44, 1.31	0.31	
Times of consuming dairy products per day (Mean/SD)^d^					0.79	0.64, 0.99		0.04
Q1 (Lowest)	87 (14.9)	1.00			1.00			
Q2	54 (10.2)	0.64	0.45, 0.93	0.02	0.68	0.46, 1.01	0.06	
Q3 (Highest)	36 (9.2)	0.58	0.38, 0.88	0.01	0.70	0.45, 1.09	0.11	
Times of consuming egg per day (Mean/SD)^d^					0.76	0.62, 0.95		0.01
Q1 (Lowest)	76 (14.5)	1.00			1.00			
Q2	63 (11.2)	0.74	0.52, 1.06	0.11	0.79	0.54, 1.15	0.22	
Q3 (Highest)	39 (9.3)	0.61	0.40, 0.92	0.02	0.60	0.38, 0.93	0.02	
Meal frequency in 24 h								–
Two times	100 (15.1)	1.00			1.00			
Three times and four times	76 (9.1)	0.56	0.41, 0.77	< 0.001	0.68	0.48, 0.96	0.03	

^a^The adjusted model included all the variables in the table except for dietary variables. And then, each of the dietary variables were put in the adjusted model above one at a time. The *p* values for trend were calculated by treating the factors as ordinal variables in the adjusted models above

^b^Parents’ age was categorized by its tertiles

^c^Puberty development was defined by the Tanner stages

^d^The frequency of consuming foods was converted into continuous variables, namely times per day, which were then categorized by its tertiles. Flesh foods included meat, poultry and fish

In terms of dietary intake, adolescents who were in the highest tertile of daily egg consumption had reduced odds (OR 0.60, 95% CI 0.38, 0.93) of having anemia as compared to those in the lowest tertile. We also observed that being in the middle tertile of flesh foods intake relative to the lowest was associated with a 0.58 (95% CI 0.38, 0.89) times lower odds of anemia. Similarly, the results for dairy products approached significance with an adjusted OR 0.68 (95% CI 0.46, 1.01). In addition, having a meal frequency of three times or more compared with two times per day was also associated with reduced odds (OR 0.68, 95% CI 0.48, 0.96) of having anemia.

We examined the associations between factors and hemoglobin concentrations in adolescents (Table 3). Similar influencing factors of anemia were identified. Besides, mothers with the non-farmer occupation, not experiencing diseases in the prior 2 weeks and stature above average were also significantly associated with higher hemoglobin concentrations in adolescents.

**Table 3:** Factors associated with hemoglobin concentrations (g/L) in adolescents from rural Western China, 2016 (*N* = 1517)

	Mean (SD)	Unadjusted			Adjusted^a^		
		Mean differences	95% CI	*p* values	Mean differences	95% CI	*p* values
Maternal age/years^b^							
Q1: ≤35	131.9 (15.1)	Ref.			Ref.		
Q2: 36–39	133.6 (14.1)	1.65	−0.20, 3.49	0.08	0.54	−1.48, 2.56	0.60
Q3: ≥40	134.0 (15.3)	2.06	0.29, 3.83	0.02	1.63	−1.26, 4.52	0.27
Maternal education							
< 3 years	129.3 (15.9)	Ref.			Ref.		
Primary	131.9 (15.5)	2.58	−0.84, 6.00	0.14	3.28	−0.24, 6.79	0.07
Secondary	133.2 (15.1)	3.86	0.56, 7.15	0.02	4.34	0.68, 8.01	0.02
High school+	135.8 (12.1)	6.49	2.83, 10.14	0.001	4.83	0.44, 9.22	0.03
Maternal occupation							
Farmer	135.0 (14.1)	Ref.			Ref.		
Others	131.8 (15.3)	3.21	1.68, 4.74	< 0.001	2.44	0.62, 4.27	0.01
Paternal age/year^b^							
Q1: ≤37	132.0 (13.9)	Ref.			Ref.		
Q2: 38–41	133.6 (15.7)	1.60	−0.21, 3.41	0.08	0.84	−1.16, 2.83	0.41
Q3: ≥42	133.6 (15.3)	1.68	−0.14, 3.51	0.07	0.57	−2.46, 3.59	0.71
Paternal education							
< 3 years	126.9 (14.9)	Ref.			Ref.		
Primary	132.3 (16.8)	5.41	−1.56, 12.39	0.13	6.05	−0.94, 13.03	0.09
Secondary	132.6 (15.3)	5.70	−1.06, 12.46	0.10	4.98	−1.92, 11.88	0.16
High school+	134.6 (12.6)	7.73	0.88, 14.59	0.03	4.25	−2.89, 11.39	0.24
Paternal occupation							
Farmer	133.3 (13.50	Ref.			Ref.		
Others	132.9 (17.3)	0.39	−1.22, 1.99	0.64	−1.50	−3.29, 0.29	0.10
Household wealth							
Low	131.6 (15.7)	Ref.			Ref.		
Medium	131.9 (16.0)	0.37	−1.50, 2.24	0.70	0.46	−1.42, 2.35	0.63
High	135.3 (12.8)	3.71	1.93, 5.48	< 0.001	2.52	0.53, 4.50	0.01
Randomized regimens							
Folic acid	133.4 (15.7)	Ref.			Ref.		
Iron/folic acid	132.4 (13.7)	−0.96	−2.78, 0.86	0.30	−0.78	−2.59, 1.02	0.39
Multiple micronutrients	133.2 (15.3)	−0.22	−2.05, 1.62	0.82	−0.28	−2.10, 1.53	0.76
Adolescent age							
10	131.2 (14.5)	Ref.			Ref.		
11	131.0 (15.7)	−0.24	−3.86, 3.81	0.90	1.08	−2.59, 4.76	0.56
12	134.0 (13.9)	2.75	−0.87, 6.37	0.14	3.05	−0.74, 6.84	0.12
13–14	135.0 (14.7)	3.78	0.06, 7.51	0.05	3.01	−1.05, 7.08	0.15
Sex							
Male	134.9 (15.2)	Ref.			Ref.		
Female	130.5 (14.1)	−4.44	−5.94, −2.94	< 0.001	−5.04	−6.64, −3.45	< 0.001
Height for age z score							
Stunting (<−2SD)	127.7 (13.5)	−4.31	−9.48, 0.86	0.10	−2.21	−7.51, 3.08	0.41
-2 to 1 SD	132.0 (14.5)	Ref.			Ref.		
Above average (>1SD)	137.6 (15.7)	5.55	3.69, 7.42	< 0.001	2.96	0.92, 5.00	0.004
Whether having an illness in the last 2 weeks							
Yes	131.7 (13.8)	Ref.			Ref.		
No	133.9 (15.4)	2.14	0.61, 3.67	0.01	1.58	0.06, 3.10	0.04
Puberty development^c^							
Pre-puberty	128.7 (14.2)	Ref.			Ref.		
Mild	132.7 (14.8)	3.95	1.83, 6.08	< 0.001	3.26	1.07, 5.44	0.004
Above mild	135.3 (14.9)	6.53	4.39, 8.68	< 0.001	5.83	3.27, 8.39	< 0.001
Times of consuming flesh foods per day (Mean/SD)^d^							
Q1 (Lowest)	130.9 (15.7)	Ref.			Ref.		
Q2	133.7 (13.5)	2.79	0.82, 4.76	0.01	2.28	0.31, 4.24	0.02
Q3 (Highest)	133.7 (15.4)	2.73	0.83, 4.64	0.01	1.31	−0.64, 3.26	0.19
Times of consuming beans per day (Mean/SD)^d^							
Q1 (Lowest)	132.7 (15.3)	Ref.			Ref.		
Q2	132.6 (14.9)	−0.04	−1.73, 1.64	0.96	−0.87	−2.56, 0.82	0.31
Q3 (Highest)	134.3 (14.2)	1.64	0.56, 3.84	0.14	0.27	−1.93, 2.47	0.81
Times of consuming dairy products per day (Mean/SD)^d^							
Q1 (Lowest)	132.2 (15.6)	Ref.			Ref.		
Q2	133.3 (14.2)	1.13	−0.62, 2.87	0.21	0.81	−0.94, 2.56	0.37
Q3 (Highest)	133.7 (14.7)	1.50	−0.41, 3.41	0.12	0.85	−1.06, 2.77	0.38
Times of consuming egg per day (Mean/SD)^d^							
Q1 (Lowest)	131.3 (14.2)	Ref.			Ref.		
Q2	134.2 (14.7)	2.86	1.14, 4.58	0.001	2.64	0.94, 4.34	0.002
Q3 (Highest)	133.4 (14.5)	2.11	0.25, 3.97	0.03	2.45	0.62, 4.29	0.01
Meal frequency in 24 h							
Two times	131.1 (15.7)	Ref.			Ref.		
Three times and four times	134.5 (14.1)	3.39	1.88, 4.90	< 0.001	1.69	0.13, 3.25	0.03

^a^The adjusted model included all the variables in the table except for dietary variables. And then, each of the dietary variables were put in the adjusted model above one at a time

^b^Parents’ age was categorized by its tertiles

^c^Puberty development was defined by the Tanner stages

^d^The frequency of consuming foods was converted into continuous variables namely times per day, which were then categorized by its tertiles. Flesh foods included meat, poultry and fish

#### Stratified analysis by sex

In addition, we found that adolescent sex modified the associations of stages of puberty and maternal education with anemia with *P* values for interaction of 0.04 and 0.01, respectively (Table 4). The statistical association between higher stages of puberty and reduced odds of anemia was achieved only among males (OR 0.35, 95% CI 0.15, 0.83) but not among females (OR 0.72, 95% CI 0.29, 1.78). Similar results for continuous hemoglobin concentrations were presented in Supplementary Table 1 (see Additional file 1).

**Table 4:** Factors associated with adolescent anemia stratified by adolescent sex in rural western China, 2016

Factors	Male			Female			*P* values for interaction between sex and factors^b^
	No. (%) of anemia	Adjusted OR^a^	95% CI	No. (%) of anemia	Adjusted OR^a^	95% CI	
Maternal age/years^c^							0.01
Q1: ≤35	37 (11.4)	1.00		47 (14.9)	1.00		
Q2: 36–39	15 (6.3)	0.54	0.24, 1.19	30 (17.9)	1.17	0.64, 2.14	
Q3: ≥40	33 (10.8)	1.01	0.39, 2.64	16 (10.0)	0.54	0.20, 1.48	
Maternal education							0.01
< 3 years	5 (8.9)	1.96	0.37, 10.44	12 (38.7)	5.84	1.51, 22.54	
Primary	39 (14.5)	3.14	0.87, 11.31	22 (13.4)	0.90	0.30, 2.70	
Secondary	37 (8.9)	1.89	0.56, 6.38	52 (15.3)	1.44	0.56, 3.68	
High school+	4 (3.1)	1.00		7 (6.7)	1.00		
Maternal occupation							0.61
Farmer	25 (7.2)	1.00		23 (9.4)	1.00		
Others	60 (11.4)	1.03	0.56, 1.90	70 (17.6)	0.58	0.33, 1.06	
Paternal age/years^c^							0.35
Q1: ≤37	30 (10.5)	1.00		41 (14.3)	1.00		
Q2: 38–41	23 (8.4)	0.83	0.40, 1.74	35 (17.2)	1.02	0.57, 1.84	
Q3: ≥42	32 (10.3)	0.73	0.26, 2.07	17 (11.2)	0.72	0.25 2.04	
Paternal education							0.48
< 3 years	2 (16.7)	1.00		2 (28.6)	1.00		
Primary	14 (10.1)	0.32	0.05, 2.01	18 (22.5)	0.79	0.12, 5.38	
Secondary	55 (10.6)	0.52	0.09, 3.18	59 (15.7)	0.56	0.08, 3.68	
High school+	14 (6.9)	0.68	0.10, 4.65	14 (7.8)	0.43	0.06, 3.17	
Paternal occupation							0.09
Farmer	42 (7.6)	1.00		56 (13.6)	1.00		
Others	40 (13.5)	0.58	0.33, 1.02	32 (15.2)	1.22	0.71, 2.12	
Household wealth							0.40
Low	39 (12.6)	1.00		36 (16.7)	1.00		
Medium	30 (11.7)	0.92	0.53, 1.61	40 (21.2)	1.62	0.93, 2.85	
High	16 (5.2)	0.56	0.27, 1.13	17 (7.1)	0.61	0.30, 1.23	
Randomized regimens							0.09
Folic acid	30 (9.6)	1.00		25 (11.6)	1.00		
Iron/folic acid	32 (11.4)	1.16	0.65, 2.07	30 (13.6)	1.10	0.59, 2.08	
Multiple micronutrients	23 (8.2)	0.95	0.52, 1.73	38 (18.4)	1.92	1.06, 3.47	
Adolescent age							0.39
10	4 (11.1)	1.00		2 (5.4)	1.00		
11	31 (10.0)	0.62	0.19, 2.02	24 (10.5)	1.61	0.35, 7.54	
12	29 (9.4)	0.75	0.23, 2.51	42 (17.6)	2.53	0.53, 12.08	
13–14	21 (9.6)	0.97	0.27, 3.52	25 (17.9)	2.67	0.52, 13.79	
Height for age z score							0.56
Stunting (<−2SD)	3 (18.8)	1.00	0.20, 5.06	4 (25.0)	2.14	0.58, 7.94	
-2 to 1 SD	73 (11.1)	1.00		80 (15.2)	1.00		
Above average (>1SD)	9 (4.5)	0.57	0.25, 1.30	9 (9.1)	0.98	0.43, 2.23	
Whether having an illness in the last 2 weeks							0.27
Yes	41 (12.2)	1.00		40 (15.8)	1.00		
No	42 (7.9)	0.63	0.39, 1.02	53 (13.6)	0.92	0.56, 1.50	
Puberty development^d^							0.04
Pre-puberty	29 (15.9)	1.00		11 (14.1)	1.00		
Mild	40 (9.5)	0.59	0.33, 1.06	31 (13.7)	0.92	0.39, 2.13	
Above mild	15 (5.7)	0.35	0.15, 0.83	51 (15.1)	0.72	0.29, 1.78	
Times of consuming flesh foods per day (Mean/SD)^e^							0.57
Q1 (Lowest)	31 (14.8)	1.00		31 (18.8)	1.00		
Q2	22 (7.9)	0.51	0.27, 0.97	32 (13.2)	0.68	0.37, 1.24	
Q3 (Highest)	32 (8.3)	0.58	0.33, 1.05	30 (12.7)	0.96	0.52, 1.77	
Times of consuming beans per day (Mean/SD)^e^							0.36
Q1 (Lowest)	33 (11.5)	1.00		29 (12.9)	1.00		
Q2	42 (10.2)	0.94	0.56, 1.59	52 (16.7)	1.55	0.90, 2.68	
Q3 (Highest)	10 (6.0)	0.54	0.24, 1.21	12 (11.8)	0.94	0.42, 2.10	
Times of consuming dairy products per day (Mean/SD)^e^							0.63
Q1 (Lowest)	44 (13.0)	1.00		43 (17.6)	1.00		
Q2	20 (6.9)	0.56	0.31, 1.02	34 (14.1)	0.80	0.46, 1.39	
Q3 (Highest)	20 (8.4)	0.70	0.38, 1.28	16 (10.5)	0.67	0.34, 1.32	
Times of consuming egg per day (Mean/SD)^e^							0.22
Q1 (Lowest)	34 (11.6)	1.00		42 (18.1)	1.00		
Q2	26 (8.3)	0.70	0.39, 1.26	37 (14.9)	0.87	0.52, 1.51	
Q3 (Highest)	25 (9.5)	0.75	0.41, 1.37	14 (8.9)	0.46	0.23, 0.92	
Meal frequency in 24 h							0.80
Two times	50 (13.1)	1.52	0.91, 2.54	50 (17.7)	1.53	0.91, 2.55	
Three times and four times	33 (6.9)	1.00		43 (12.1)	1.00		

^a^The adjusted model included all the variables in the table except for dietary variables. And then, each of the dietary variables were put in the adjusted model above one at a time

^b^The p values for interaction between sex and factors were calculated using likelihood-ratio test between including interaction terms and not including in the models

^c^Parents’ age was categorized by its tertiles

^d^Puberty development was defined by the Tanner stages

^e^The frequency of consuming foods was converted into continuous variables namely times per day, which were then categorized by its tertiles. Flesh foods included meat, poultry and fish

## Discussion

We found that the overall prevalence of anemia in our study population of adolescents aged 10–14 years in rural western China was 11.7%, with a prevalence of 9.7 and 14.4% among adolescent males and females, respectively. Multivariable analysis identified that lower maternal education, lower household wealth, female sex, pre-puberty development, lower consumption of flesh foods, eggs and dairy products, and lower meal frequency per day were associated with an increased likelihood of anemia. Moreover, we found that a higher stage of puberty was statistically associated with a reduced risk of anemia for male adolescents but not for females.

The prevalence of anemia in our study population (11.7%) would be classified as a mild public health problem according to the WHO [15]. The prevalence is also lower than the prevalence of 25.5% from a cross-sectional survey among middle-school students conducted in the study area conducted in 2006 [34]. Similar decrements in anemia prevalence have also been observed in other rural areas in western China [35, 36]. Our findings further support that the prevalence of adolescent anemia in China has improved to some extent. Nevertheless, the prevalence of adolescent anemia in our study population was still higher than that in more developed areas of China such as 2.9% among adolescents aged 12–14 in Yiwu, a city in eastern China [37], which warrants the development of intervention strategies for adolescent anemia in rural western China.

We found positive associations between being female and adolescent anemia which was in line with previous studies conducted in Indonesia and Turkey [9, 18]. This finding may be explained by the occurrence of menarche and regular blood loss [8]. Further differences in diet may also be a contributor. One study from India reported that adolescent girls tended to consume fewer protein- and vitamin-rich foods as compared with boys [38].

In addition, we found that males with higher stages of puberty development had reduced odds of anemia while the statistical association was not achieved among females, which was similar to the results of a study from Indonesia [9]. Some studies have reported that hemoglobin concentrations in adolescent boys may increase with the onset of puberty and may be attributed to testosterone and other androgen effects [39, 40]. However, our study cannot provide evidence for the causality between puberty development and anemia due to the cross-sectional design. Based on a cohort study in Pakistan, Campisi and colleagues reported that anemia and stunting in childhood may delay the onset of adolescent puberty [41]. As a result, further research is needed on the relationship of puberty and anemia among adolescents in LMICs.

Some studies have reported that the risk of having anemia increases with age among adolescents that may be explained by puberty development [13], however these studies did not adjust for stages of puberty. We noted significant associations between continuous adolescent age and anemia even after adjusting for stages of puberty. Further, the models produced a condition index of 8.16 which indicated that the multicollinearity between age and stages of puberty was not a concern (data not shown) [42]. We noted, in the same study population, that adolescent age was positively associated with the prevalence of stunting. Taken together, these findings suggest that data on adolescent health outcomes e.g., anemia, should be reported by sex and age and that when possible stages of puberty should be factored in the interpretation of findings.

Although early adolescence is a critical period in the dietary transition from mid-childhood through adolescence to adulthood [43, 44], dietary data on anemia in this age group are limited. In the adjusted models, we found that higher consumption of eggs, flesh foods and dairy products was associated with decreased likelihood of anemia. A meta-analysis of randomized controlled trials in China also reported that dietary interventions such as consuming eggs may significantly reduce the risk of iron deficiency among children with iron-deficiency anemia [45]. Further, a study conducted in a refugee camp in Ethiopia found that adolescent girls who consumed greater heme-iron containing food sources were less likely to be anemic [13]. Foods such as eggs, dairy products, and flesh foods, are important sources of protein, vitamin B_12_, bioavailable iron and other micronutrients which may influence the risk of anemia [46]. In a recent study, it was estimated that less than half of adolescent girls consumed dairy products, flesh foods, or eggs on a daily basis (41, 46, and 19%, respectively) in LMICs [47]. WHO recommends weekly iron and folic acid supplementation for menstruating adolescent girls in settings with 20% or higher levels of anemia prevalence [32], however whether this public health program should be extended to adolescent boys remains unclear. In agreement with a study in southern Ethiopia [48], we found that consuming three or more of meals per day was associated with reduced risk of anemia. We hypothesize that adolescents who eat three or more meals per day have a higher likelihood of meeting their nutritional requirements. It is notable that in our study population majority of participants only had two meals per day and ate the first meal at noon and skipped the breakfast. One study from China reported that skipping breakfast was associated with higher risk of stunting, wasting and malnutrition among children aged 6–17 years [49].

In addition to biomedical influences, we also found that adolescents from higher-income households and those whose mothers had higher education levels were less likely to be anemic, which is in accord with other studies [6, 11]. Individuals from higher socioeconomic status may have access and consume more iron- and vitamin C-rich foods [6]. Tur and colleagues also reported that maternal education was positively associated with the quality of dietary intake of mineral and vitamin among adolescents [50]. In the stratified analysis by sex, the association of maternal education with adolescent anemia was only found in females but not in males. We hypothesize that adolescent girls are more likely to follow maternal advice on healthy behaviors as compared to boys [51]; however, this hypothesis warrants further study. Therefore, programmes that only emphasize biomedical factors might not be sufficient to prevent adolescent anemia.

This study has limitations that should be noted. First, the study included adolescents who were born to women who participated in an antenatal micronutrient supplementation trial, and this population may not be a truly representative sample of our target population of adolescents in rural western China. Our prior data had shown that the background characteristics between participants followed and those lost to follow-up at adolescence were balanced [28]. Besides, the cluster-randomized trial included all pregnant women in villages, representing the community to some extent. Second, due to no available data on older adolescents in the present study, we were not able to compare the potential differences in the prevalence and risk factors for anemia between early adolescence and older adolescence. Third, we focused on high protein-, vitamin-, and mineral-based foods, but some studies reported that regular consumptions of fruits and green leafy vegetables were also associated with reduced odds of having anemia in adolescent girls [52], and this is an area to pursue in further research. In addition, other factors associated with anemia were not accounted in the present study such as parasite infection, thalassemia, and maternal nutritional status. Finally, owing to the cross-sectional study design, prospective studies are needed to verify the relationships between factors and anemia among adolescents. Our results, however, provide evidence that can help develop intervention strategies and target at high-risk adolescent populations.

## Conclusions

We found that anemia was a mild public health problem among young adolescents aged 10–14 years in rural western China. Integrated interventions that address biomedical determinants and targeting high-risk populations of adolescents may be essential to reduce the risk of anemia and improve health among this critical age group.

## Supplementary Information

**Additional file 1: Supplementary Table 1.** Factors associated with adolescent hemoglobin concentrations (g/L) stratified by adolescent sex in rural western China, 2016.

## Data Availability

All data generated or analyzed during this study are included in this published article and its supplementary information files. Data sharing is available from the corresponding author on reasonable request.

## Abbreviations

BAZ: Body mass index for-age z score
CI: Confidence interval
HAZ: Height for-age z score
Hb: Hemoglobin
LMICs: Low- and middle-income countries
OR: Odds ratio
SD: Standard deviation
WHO: World Health Organization

## References

[1] Fuhrmann D, Knoll LJ, Blakemore S (2015). Adolescence as a sensitive period of brain development. Trends Cogn Sci.

[2] Christian P, Smith ER (2018). Adolescent undernutrition: global burden, physiology, and nutritional risks. Ann Nutr Metab.

[3] Beard JL (2001). Iron biology in immune function, muscle metabolism and neuronal functioning. J Nutr.

[4] Pivina L, Semenova Y, Doşa MD, Dauletyarova M, Bjørklund G (2019). Iron deficiency, cognitive functions, and neurobehavioral disorders in children. J Mol Neurosci.

[5] Bhutta ZA, Lassi ZS, Bergeron G, Koletzko B, Salam R, Diaz A, McLean M, Black RE, De-Regil LM, Christian P (2017). Delivering an action agenda for nutrition interventions addressing adolescent girls and young women: priorities for implementation and research. Ann N Y Acad Sci.

[6] Kim JY, Shin S, Han K, Lee KC, Kim JH, Choi YS, Kim DH, Nam GE, Yeo HD, Lee HG (2014). Relationship between socioeconomic status and anemia prevalence in adolescent girls based on the fourth and fifth Korea National Health and nutrition examination surveys. Eur J Clin Nutr.

[7] Mistry SK, Jhohura FT, Khanam F, Akter F, Khan S, Yunus FM, Hossain MB, Afsana K, Haque MR, Rahman M (2017). An outline of anemia among adolescent girls in Bangladesh: findings from a cross-sectional study. BMC Hematol.

[8] Sabale R, Kowli S, Chowdary P (2013). Prevalence of anemia and its determinants in urban school-going children of Mumbai. Int J Med Public Health.

[9] Soekarjo DD, de Pee S, Bloem MW, Tjiong R, Yip R, Schreurs WH (2001). Muhilal. Socio-economic status and puberty are the main factors determining anaemia in adolescent girls and boys in East Java, Indonesia. Eur J Clin Nutr.

[10] Sekhar DL, Murray-Kolb LE, Kunselman AR, Weisman CS, Paul IM (2016). Differences in risk factors for anemia between adolescent and adult women. J Women's Health.

[11] Mchiza ZJ, Parker W, Sewpaul R, Job N, Chola L, Mutyambizi C, Sithole M, Stokes A, Labadarios D (2018). Understanding the determinants of hemoglobin and iron status: adolescent-adult women comparisons in SANHANES-1. Ann N Y Acad Sci.

[12] Cole TJ, Bellizzi MC, Flegal KM, Dietz WH (2000). Establishing a standard definition for child overweight and obesity worldwide: international survey. BMJ..

[13] Engidaw MT, Wassie MM, Teferra AS (2018). Anemia and associated factors among adolescent girls living in aw-Barre refugee camp, Somali regional state, Southeast Ethiopia. PLoS One.

[14] Teji K, Dessie Y, Assebe T, Abdo M (2016). Anaemia and nutritional status of adolescent girls in Babile District, Eastern Ethiopia. Pan Afr Med J.

[15] WHO (2001). Iron deficiency anaemia: Assessment, prevention, and control-A guide for programme managers.

[16] Haerens L, Vereecken C, Maes L, De Bourdeaudhuij I (2010). Relationship of physical activity and dietary habits with body mass index in the transition from childhood to adolescence: a 4-year longitudinal study. Public Health Nutr.

[17] Biazzi Leal D, Altenburg De Assis MA, Hinnig PDF, Schmitt J, Soares Lobo A, Bellisle F, Di Pietro P, Vieira F, de Moura Araujo PH, de Andrade D (2017). Changes in dietary patterns from childhood to adolescence and associated body adiposity status. Nutrients.

[18] Isik BY, Karabulut A, Gurses D, Ethem CI (2012). Prevalence and risk factors of anemia among adolescents in Denizli, Turkey. Iran J Pediatr.

[19] Gonete KA, Tariku A, Wami SD, Derso T (2018). Prevalence and associated factors of anemia among adolescent girls attending high schools in Dembia District, Northwest Ethiopia, 2017. Arch Public Health.

[20] Tesfaye M, Yemane T, Adisu W, Asres Y, Gedefaw L (2015). Anemia and iron deficiency among school adolescents: burden, severity, and determinant factors in Southwest Ethiopia. Adolesc Health Med Ther.

[21] Ahankari AS, Myles PR, Fogarty AW, Dixit JV, Tata LJ (2017). Prevalence of iron-deficiency anaemia and risk factors in 1010 adolescent girls from rural Maharashtra, India: a cross-sectional survey. Public Health.

[22] Chang JL, Wang Y (2016). Chinese nutrition and health surveillance-the comprehensive report between 2010–2013.

[23] Li M, Hu Y, Mao D, Wang R, Chen J, Li W, Yang X, Piao J, Yang L (2017). Prevalence of Anemia among Chinese rural residents. Nutrients..

[24] Wu J, Hu Y, Li M, Chen J, Mao D, Li W, Wang R, Yang Y, Piao J, Yang L (2019). Prevalence of anemia in Chinese children and adolescents and its associated factors. Int J Env Res Pubic Health.

[25] Luo D, Yan X, Hu P, Zhang J, Lei Y, Song Y, Ma J (2019). Subnational disparity of anemia among Chinese Han students aged 7-14 years in 2014. Chin J Sch Health.

[26] Luo D, Xu R, Ma J, Yan X, Hu P, Song Y, Jan C, Raat H, Patton GC (2020). The associations of economic growth and anaemia for school-aged children in China. Matern Child Nutr.

[27] Zeng L, Dibley MJ, Cheng Y, Dang S, Chang S, Kong L, Yan H (2008). Impact of micronutrient supplementation during pregnancy on birth weight, duration of gestation, and perinatal mortality in rural western China: double blind cluster randomised controlled trial. BMJ..

[28] Zhu Z, Cheng Y, Zeng L, Elhoumed M, He G, Li W, Zhang M, Li W, Li D, Tsegaye S (2018). Association of antenatal micronutrient supplementation with adolescent intellectual development in rural western China. JAMA Pediatr.

[29] Butte NF, Garza C, de Onis M (2007). Evaluation of the feasibility of international growth standards for school-aged children and adolescents. J Nutr.

[30] Marshall WA, Tanner JM (1969). Variations in pattern of pubertal changes in girls. Arch Dis Child.

[31] Marshall WA, Tanner JM (1970). Variations in the pattern of pubertal changes in boys. Arch Dis Child.

[32] Filmer D, Pritchett LH (2001). Estimating wealth effects without expenditure data--or tears: an application to educational enrollments in states of India. Demography..

[33] World Health Organization (2011). Haemoglobin concentrations for the diagnosis of anaemia and assessment of severity.

[34] Wang X, Wang Z, Xiao S, Zhang J, Lei Y (2008). Analysis of anaemic causes among students of two middle schools in Guanzhong rural regions of Shaanxi province. J Xi’an Jiaotong Univ (Med Sci).

[35] Luo R, Kleiman-Weiner M, Rozelle S, Zhang L, Liu C, Sharbono B, Shi Y, Yue A, Martorell R, Lee M (2010). Anemia in rural China's elementary schools: prevalence and correlates in Shaanxi Province's poor counties. Ecol Food Nutr.

[36] Luo R, Zhang L, Liu C, Zhao Q, Shi Y, Miller G, Yue E, Sharbono B, Medina A, Rozelle S (2011). Anaemia among students of rural China's elementary schools: prevalence and correlates in Ningxia and Qinghai's poor counties. J Health Popul Nutr.

[37] Sun C, Zhang R, Zhu Z, Dong X, Huang K, Huang L, Chen L, Li Y, Zong S (2018). Nutritional status among children and adolescents in Yiwu. Prev Med.

[38] Aurino E (2017). Do boys eat better than girls in India? Longitudinal evidence on dietary diversity and food consumption disparities among children and adolescents. Econ Hum Biol.

[39] Thomsen K, Riis B, Krabbe S, Christiansen C (1986). Testosterone regulates the haemoglobin concentration in male puberty. Acta Paediatr Scand.

[40] Hero M, Wickman S, Hanhijarvi R, Siimes MA, Dunkel L (2005). Pubertal upregulation of erythropoiesis in boys is determined primarily by androgen. J Pediatr.

[41] Campisi SC, Humayun KN, Rizvi A, Lou W, Söder O, Bhutta ZA (2019). Later puberty onset among chronically undernourished adolescents living in a Karachi slum, Pakistan. Acta Paediatr.

[42] Belsley DA (1991). Conditioning diagnostics, collinearity and weak data in regression.

[43] Winpenny EM, van Sluijs EMF, White M, Klepp K, Wold B, Lien N (2018). Changes in diet through adolescence and early adulthood: longitudinal trajectories and association with key life transitions. Int J Behav Nutr Phy.

[44] Winpenny EM, Penney TL, Corder K, White M, van Sluijs EMF (2017). Change in diet in the period from adolescence to early adulthood: a systematic scoping review of longitudinal studies. Int J Behav Nutr Phy.

[45] Sun J, Zhang L, Cui J, Li S, Lu H, Zhang Y, Li H, Sun J, Baloch Z (2018). Effect of dietary intervention treatment on children with iron deficiency anemia in China: a meta-analysis. Lipids Health Dis.

[46] USAID (2013). Conceptual frameworks for anemia.

[47] Keats EC, Aviva IR, Reena J, Christina OH, Shailja S, Zulfiqar AB (2018). Diet and eating practices among adolescent girls in low- and middle-income countries: A systemic review.

[48] Shaka MF, Wondimagegne YA (2018). Anemia, a moderate public health concern among adolescents in South Ethiopia. PLoS One.

[49] Li L, Xu P, Yang T, Gan Q, Cao W, Pan H, Xu J, Hu X, Zhang Q (2018). Relationship between breakfast and nutrition status study of children aged 6-17 in China from 2010 to 2012. Wei Sheng Yan Jiu.

[50] Tur JA, Puig MS, Benito E, Pons A (2004). Associations between sociodemographic and lifestyle factors and dietary quality among adolescents in Palma de Mallorca. Nutrition..

[51] Greene A, Michelle GD (1990). Age and gender differences in adolescents’ preferences for parental advice: Mum's the word. J Adolesc Res.

[52] Ghatpande NS, Apte PP, Naik SS, Kulkarni PP (2019). Fruit and vegetable consumption and their association with the indicators of iron and inflammation status among adolescent girls. J Am Coll Nutr.

