# Supplementary material for: Anemia and associated factors among adolescent girls and boys at 10–14 years in rural western China
Source: BMC Public Health. 2021 Jan 26;21:218. doi: 10.1186/s12889-021-10268-z (PMC7836596; doi:10.1186/s12889-021-10268-z)
Supplement: Supplementary file 1 — Additional file 1: Supplementary Table 1. Factors associated with adolescent hemoglobin concentrations (g/L) stratified by adolescent sex in rural western China, 2016. [file 12889_2021_10268_MOESM1_ESM.docx]

Supplementary Table 1 Factors associated with adolescent hemoglobin concentrations (g/L) stratified by adolescent sex in rural western China, 2016

| Factors | Male | | Female | | P values for interaction between sex and factors^b^ |
| --- | --- | --- | --- | --- | --- |
|  | Mean (SD) | Adjusted mean differences (95% CI) ^a^ | Mean (SD) | Adjusted mean differences (95% CI)^a^ |  |
| Maternal age/years^c^ |  |  |  |  | 0.62 |
| Q1: ≤35 | 133.7(15.9) | Ref. | 130.2(14.0) | Ref. |  |
| Q2: 36-39 | 135.5(13.6) | 1.00(-1.82, 3.83) | 130.8(14.3) | 0.10(-2.77, 2.97) |  |
| Q3: ≥40 | 135.7(15.6) | 2.08(-1.79, 5.95) | 130.7(14.1) | 1.70(-2.64, 6.05) |  |
| Maternal education |  |  |  |  | 0.003 |
| < 3 years | 134.2(12.4) | Ref. | 120.5(17.7) | Ref. |  |
| Primary | 132.7(16.3) | -0.36(-4.84, 4.11) | 130.5(13.9) | 11.95(6.23, 17.68) |  |
| Secondary | 135.3(15.3) | 2.09(-2.60, 6.79) | 130.5(14.6) | 10.73(4.81, 16.64) |  |
| High school+ | 138.3(12.9) | 2.16(-3.67, 7.98) | 132.7(10.2) | 11.20(4.38, 18.01) |  |
| Maternal occupation |  |  |  |  | 0.64 |
| Farmer | 133.6(15.1) | Ref. | 132.2(12.0) | Ref. |  |
| Others | 136.9(15.1) | 1.88(-0.54, 4.29) | 129.4(15.2) | 3.06(0.30, 5.83) |  |
| Paternal age/years^c^ |  |  |  |  | 0.62 |
| Q1: ≤37 | 133.6(14.2) | Ref. | 130.3(13.4) | Ref. |  |
| Q2: 38-41 | 135.6(16.2) | 1.23(-1.59, 4.06) | 130.8(14.5) | 0.60(-2.20, 3.40) |  |
| Q3: ≥42 | 135.4(15.2) | 0.35(-3.67, 4.37) | 130.1(15.0) | 0.31(-4.30, 4.92) |  |
| Paternal education |  |  |  |  | 0.22 |
| < 3 years | 132.5(12.1) | Ref. | 117.3(15.2) | Ref. |  |
| Primary | 134.5(16.3) | 2.86(-6.23, 11.95) | 128.6(17.0) | 11.70(0.84, 22.57) |  |
| Secondary | 134.3(15.6) | 1.28(-7.77, 10.34) | 130.2(14.6) | 11.28(0.65, 21.92) |  |
| High school+ | 136.7(13.5) | -0.03(-9.46, 9.40) | 132.3(11.0) | 11.68(0.75, 22.61) |  |
| Paternal occupation |  |  |  |  | 0.78 |
| Farmer | 134.6(18.1) | Ref. | 130.6(13.2) | Ref. |  |
| Others | 135.2(13.5) | -1.12(-3.49, 1.26) | 130.5(15.9) | -1.85(-4.54, 0.83) |  |
| Household wealth |  |  |  |  | 0.39 |
| Low | 133.1(15.3) | Ref. | 129.3(15.9) | Ref. |  |
| Medium | 134.1(16.7) | 1.83(-0.67, 4.33) | 129.0(14.6) | -1.00(-3.86, 1.86) |  |
| High | 137.3(13.3) | 3.12(0.45, 5.80) | 132.6(11.5) | 1.37(-1.61, 4.35) |  |
| Randomized regimens |  |  |  |  | 0.07 |
| Folic acid | 135.5(16.9) | Ref. | 130.4(13.2) | Ref. |  |
| Iron/folic acid | 133.6(13.6) | -2.30(-4.69, 0.08) | 131.0(13.7) | 0.87(-1.85, 3.60) |  |
| Multiple micronutrients | 135.6(14.7) | -0.04(-2.46, 2.37) | 130.0(15.5) | -0.78(-3.52, 1.96) |  |
| Adolescent age |  |  |  |  | 0.08 |
| 10 | 129.8(16.0) | Ref. | 132.6(12.9) | Ref. |  |
| 11 | 132.4(16.6) | 4.10(-1.03, 9.22) | 129.1(14.3) | -2.07(-7.29, 3.15) |  |
| 12 | 135.8(14.2) | 5.70(0.47, 10.95) | 131.5(13.3) | 0.76(-4.74, 6.27) |  |
| 13-14 | 138.0(13.6) | 5.94(0.38, 11.51) | 130.3(15.3) | -0.46(-6.45, 5.52) |  |
| Height for age z score |  |  |  |  | 0.03 |
| Stunting (<-2SD) | 132.3(11.9) | 0.94(-6.61, 8.48) | 123.1(13.8) | -8.08(-15.48, -0.67) |  |
| -2 to 1 SD | 133.2(14.8) | Ref. | 130.5(14.1) | Ref. |  |
| Above average (>1SD) | 140.5(15.5) | 4.08(1.47, 6.69) | 131.6(14.2) | -0.17(-3.55, 3.21) |  |
| Whether having illness in last two weeks |  |  |  |  | 0.80 |
| Yes | 133.6(14.1) | Ref. | 129.3(12.9) | Ref. |  |
| No | 135.8(15.6) | 1.78(-0.25, 3.81) | 131.2(14.8) | 1.37(-0.91, 3.66) |  |
| Puberty development |  |  |  |  | <0.001 |
| Pre-puberty | 129.1(14.6) | Ref. | 127.8(13.3) | Ref. |  |
| Mild | 133.8(14.7) | 3.70(1.04, 6.37) | 130.5(14.7) | 1.43(-2.41, 5.27) |  |
| Above mild | 140.7(14.5) | 8.51(5.16, 11.86) | 131.0(13.8) | 1.70(-2.40, 5.81) |  |
| Times of consuming flesh foods per day (Mean/SD)^d^ |  |  |  |  | 0.25 |
| Q1 (Lowest) | 133.4(15.1) | Ref. | 127.8(16.0) | Ref. |  |
| Q2 | 135.2(13.1) | 0.59(-2.10, 3.28) | 132.1(13.8) | 3.74(0.90, 6.59) |  |
| Q3 (Highest) | 135.5(16.6) | 0.56(-2.03, 3.15) | 130.6(12.7) | 1.44(-1.52, 4.39) |  |
| Times of consuming beans per day (Mean/SD)^d^ |  |  |  |  | 0.96 |
| Q1 (Lowest) | 134.4(16.0) | Ref. | 130.5(14.1) | Ref. |  |
| Q2 | 134.6(15.3) | -0.87(-3.14, 1.39) | 130.0(14.1) | -1.20(-3.71, 1.30) |  |
| Q3 (Highest) | 136.2(13.8) | 0.06(-2.81, 2.93) | 131.3(14.3) | 0.54(-2.88, 3.97) |  |
| Times of consuming dairy products per day (Mean/SD)^d^ |  |  |  |  | 0.66 |
| Q1 (Lowest) | 133.8(15.9) | Ref. | 130.1(15.0) | Ref. |  |
| Q2 | 135.8(14.5) | 1.44(-0.90, 3.78) | 130.4(13.2) | -0.19(-2.80, 2.42) |  |
| Q3 (Highest) | 135.4(14.8) | 1.18(-1.30, 3.67) | 131.1(14.1) | 0.20(-2.77, 3.17) |  |
| Times of consuming egg per day (Mean/SD)^d^ |  |  |  |  | 0.17 |
| Q1 (Lowest) | 133.0(13.9) | Ref. | 129.1(14.4) | Ref. |  |
| Q2 | 136.7(14.9) | 3.65(1.42, 5.88) | 130.9(13.9) | 1.46(-1.12, 4.04) |  |
| Q3 (Highest) | 134.3(14.6) | 2.02(-0.31, 4.35) | 131.8(14.2) | 2.99(0.08, 5.91) |  |
| Meal frequency in 24 hours |  |  |  |  | 0.33 |
| Two times | 133.0(16.1) | Ref. | 128.5(14.7) | Ref. |  |
| Three times and four times | 136.4(14.2) | 0.65(-1.42, 2.72) | 132.0(13.5) | 2.75(0.37, 5.12) |  |

^a^The adjusted model included all the variables in the table except for dietary variables. And then, each of the dietary variables were put in the adjusted model above one at a time.

^b^The p values for interaction between sex and factors were calculated using likelihood-ratio test between including interaction terms and not including in the models.

^c^Parents’ age was categorized by its tertiles.

^d^The frequency of consuming foods was converted into continuous variables namely times per day, which were then categorized by its tertiles. Flesh foods included meat, poultry and fish.
